# Transthyretin Amyloidosis—From Peculiar Neuropathy to a Treatable Chronic Multisystemic Disease

**DOI:** 10.3390/genes17060680

**Published:** 2026-06-10

**Authors:** Sasha A. Živković, J. David Avila

**Affiliations:** 1 Department of Neurology, School of Medicine, Yale University, New Haven, CT 06510, USA; 2 Department of Neurology, Geisinger Medical Center, Danville, PA 17821, USA; jdavila1@geisinger.edu

**Keywords:** amyloidosis, transthyretin, amyloid cardiomyopathy, amyloid neuropathy, dysautonomia, oculoleptomeningeal amyloidosis, ATTR

## Abstract

Transthyretin amyloidosis (ATTR) is a multisystemic disorder associated with extracellular accumulation of misfolded transthyretin (TTR) protein forming insoluble amyloid deposits. Depending on the TTR genotype, ATTR is classified as hereditary ATTR (ATTRv) with pathogenic gene variants and wild-type ATTR (ATTRwt) with a normal TTR genotype. Two cardinal clinical manifestations of ATTR are amyloid cardiomyopathy and peripheral neuropathy, but multisystemic deposition of amyloid may also manifest with ocular and leptomeningeal amyloidosis, various orthopedic complications (carpal tunnel syndrome, spinal stenosis), nephropathy, and gastrointestinal and pulmonary amyloidosis. The natural history of untreated ATTR is characterized by progressive worsening and 25% of patients may die within 24 months from the onset. The first treatment for ATTR was liver transplantation which slows the disease progression, but its use was limited by the scarcity of available liver allografts and complex post-transplant morbidities associated with immunosuppression and various metabolic disturbances. Recent introduction of TTR stabilizers and gene silencing has significantly changed the outcomes and reduced ATTR-related morbidities and mortality, and early diagnosis remains important for improved outcomes. In our narrative expert review, we are discussing epidemiological and clinical features of ATTR, its pathophysiology and available treatments as rapidly progressive fatal disease is being transformed into a treatable chronic disease.

## 1. Introduction and History

Transthyretin amyloidosis (ATTR) is a multisystemic disorder associated with extracellular accumulation of misfolded transthyretin (TTR) protein forming insoluble amyloid deposits. Systemic amyloidoses are a group of multisystemic disorders caused by the accumulation of misfolded proteins that become resistant to the catabolic processes, leading to organ dysfunction [1]. The initial descriptions of amyloidosis came from autopsy cases. Nicolaus Fontanus is considered the first to pathologically describe the sago spleen of amyloidosis in 1639. Later descriptions identified the deposition of a waxy or lardaceous substance, which was thought to be fatty, in the spleen or kidneys of patients with chronic diseases such as tuberculosis and syphilis. George Budd concluded that the deposits were albuminous [2].

In 1838, the German botanist Matthias Schleiden coined the term “amyloid” to describe a normal amylaceous component of plants. Rudolf Virchow, a German pathologist and the father of modern pathology, used the term to illustrate the reaction of cerebral corpora amylacea with iodine [2].

The two most common systemic amyloidoses are immunoglobulin light chain amyloidosis and transthyretin amyloidosis (ATTR) [1]. Samuel Wilks reported the first case of primary or light chain amyloidosis in 1856. Autopsy demonstrated cardiac hypertrophy and lardaceous changes in the spleen and kidneys. In 1865, he published an additional 60 cases of “lardaceous disease” [2,3].

The first report of familial or hereditary amyloidosis was probably published by De Bruyn and Stern in 1929, in a patient with sensory symptoms, diarrhea, and a family history of the disease. Autopsy revealed deposits of a homogeneous substance in the nerves, which was thought to represent myelin. They alluded to the familial nature of the disease and attributed a previous description to Dejerine–Sottas disease [2,4].

Undoubtedly, the Portuguese neurologist Corino Andrade provided the most detailed clinical description of what he called “a peculiar form of peripheral neuropathy,” now known as hereditary transthyretin amyloid polyneuropathy. He saw the first case of what was called “mal dos pesinhos” (“Foot disease”), in a 37-year-old woman from Povoa de Varzim, a fishing town in Northern Portugal. His seminal publication in *Brain* in 1952 summarized 74 cases, including elegant clinicopathologic descriptions of two of them. He recognized multiple distinctive features of the disease, including its familial nature, early age of onset, classic phenotype, typical progression, and amyloid deposition in nerves [5].

The transthyretin protein (TTR, OMIM #176300) was identified in the early 1950s and was initially named thyroxine-binding prealbumin. It was later recognized to also transport vitamin A, so it was renamed as transthyretin [6]. In 1983, Saraiva et al. reported that in Portuguese patients with familial amyloid polyneuropathy, there was a valine substitution of methionine at position 30 of the TTR gene, which is now known to be the most common pathogenic neuropathic variant of the disease (Val30Met) [7]. In 1987, Benson et al. reported another Appalachian variant of ATTR with alanine substitution of threonine at position 60 and cardiomyopathic phenotype [8]. The TTR gene was fully sequenced in 1985 [9]. To date, more than 140 pathogenic variants have been reported [10].

This narrative expert review is based on recent key translational and clinical studies, and aims to review and update the management of ATTR. Our search was based on results from studies on ATTR found in PubMed using the combination of terms “Transthyretin amyloidosis” and “ATTR” up to 15 April 2026.

## 2. Epidemiology

Wild-type ATTR (ATTRwt) is the most common type of ATTR, and primarily manifests with cardiomyopathy. The epidemiology of ATTRv varies by region, type of amyloidosis, clinical manifestations, and population studied. Hereditary transthyretin amyloidosis (ATTRv) is a rare disease with increased prevalence of carrier status in endemic populations, including western Africans (V122I—3.4%), Portuguese and Swedes (V30M—northern Portugal—0.1%, northern Sweden—2%) and northern Irish (T60A—northern Ireland—1.1%) [11,12,13,14]. In the whole country of Portugal, the prevalence has been estimated at 22.93 per 100,000 adults [15]. The same variants can be seen sporadically in various populations worldwide.

A recent systematic literature review by Delgado et al. provides a comprehensive overview of the epidemiology of the disease [16].

In the US, the estimated prevalence of ATTR cardiomyopathy (ATTR-CA) is 6.09 per million, and the incidence was estimated at 3.96 per million person-years (PMPY) [17]. In contrast, in Japan, the prevalence of ATTR-CA has been reported to be as high as 70.3 to 86.1 per million for ATTRwt and 2.4 to 2.9 per million for ATTRv, while the incidence may be up to 100 PMPY [18,19]. Of note, the latter figure was seen in individuals 65 years or older. In Europe, prevalence was estimated between 14 and 50 per million [16].

A nationwide study of ATTRv-polyneuropathy (ATTRv-PN) in Portugal found a prevalence of 229.3 per million, and an incidence of 8.7 PMPY. Interestingly, the recent report indicated a decline in incidence while the prevalence increased compared to previous estimates [15]. A 2018 study estimated the global prevalence of ATTRv PN ranging from 5526 to 38,468 persons [20]. This is likely an underestimation, as it was performed before the widespread use of TTR silencers and expanded access to genetic testing, which has facilitated the detection of ATTRv PN.

Multiple studies have investigated the prevalence of ATTR-CA in patients with heart failure, with reported rates ranging from 0.31% to 37% [16]. While ATTRwt is much more common than ATTRv, especially with late onset ATTR-CA, a recent cohort study in a non-endemic region (United Kingdom) showed that 20.7% of elderly ATTR-CA patients (older than 70 years) had ATTRv [21]. Carpal tunnel syndrome, particularly when bilateral, has long been recognized as an early and common manifestation of amyloidosis that may precede ATTR-CA or ATTRv-PN by 5–10 years [22,23]. Studies of tenosynovial biopsies in patients undergoing carpal tunnel release have reported a wide range of prevalences of amyloid-positive tissue from 2.3% to 39% [22,24,25]. In patients with spinal stenosis, screening with ligamentum flavum biopsy has demonstrated a prevalence of 5% to 45% [26].

ATTRv is endemic in Portugal, Sweden, parts of Japan, and Brazil. In the THAOS database, V30M was the most common reported pathogenic variant worldwide, and V122I was the most common pathogenic variant in the United States and the second most common worldwide [27,28]. There was a male predominance of reported ATTR patients in the database, regardless of phenotype, with the mean age of symptom onset being 56 years [27].

## 3. Pathophysiology

**Figure 1:**
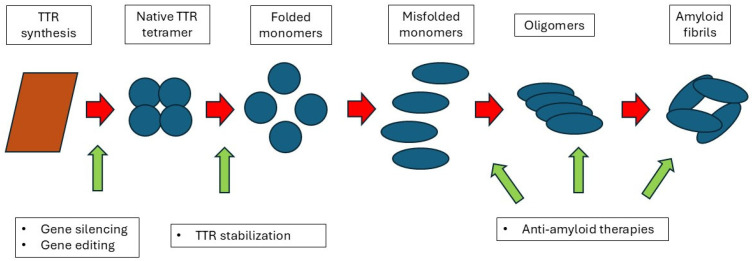
Pathogenesis of ATTR and therapeutic approaches.

Transthyretin (TTR) is a homotetrameric protein that transports retinol binding protein and thyroxine [29,30]. TTR is synthesized mostly in the liver, and also in the choroid plexus, retinal pigment epithelium and pancreas. The exact physiologic roles of TTR remain uncertain but it seems to have a neuroprotective role and may serve as a shuttling detoxifying mechanism for various molecules [29,30]. In the experimental model of TTR knockout mice, animals without TTR remain fertile and appear normal with reduced levels of serum retinol, RBP and thyroxine [31]. In TTR knockout mice, the lack of TTR may also delay sciatic nerve recovery after crush injury and lead to mild behavioral effects [30]. TTR performs its shuttling roles in its tetrameric conformation. Pathogenic variants of TTR are associated with decreased stability of TTR tetramers, leading to an increased formation of monomers. In contrast, some nonpathogenic variants (e.g., T119M) stabilize tetramers and alleviate the destabilizing effects of pathogenic TTR variants [32]. The mechanism of the TTR stabilizer acoramidis mimics the stabilizing effects of the T119M TTR variant [33]. Destabilized tetramers dissociate into dimers and monomers, followed by misfolding of monomers and their aggregation into insoluble amyloid fibrils which deposit in various tissues and organs (Figure 1) [34].

The underlying mechanisms of TTR tetramer destabilization are not completely understood but there is a role for shear stress and mechano-enzymatic proteolysis [35]. Tissue injury in ATTR is caused by extracellular accumulation of insoluble deposits of misfolded ATTR protein [34].

Pathophysiologic mechanisms of tissue injury in ATTR are not completely understood and the importance of specific mechanisms may vary in different types of tissue, including physical displacement of normal parenchymal tissue, disruption of the tissue architecture and cellular toxicity [36].

## 4. ATTR Types as Defined by TTR Genotype

Clinical types of ATTR include hereditary or variant ATTR (ATTRv), wild-type ATTR (ATTRwt) and acquired ATTR. In ATTRv, also known as hereditary ATTR, the accumulation of amyloid deposits occurs as a result of genetic variants of TTR prone to amyloid formation. In ATTRwt, the TTR genotype is normal and the exact mechanism leading to amyloid formation and ATTR in primarily elderly individuals is not well understood. Acquired ATTR affects liver allograft recipients after domino liver transplantation when a functional liver explant from individuals with ATTRv is transplanted to individuals with liver failure, later leading to ATTR.

### 4.1. Variant ATTR (Hereditary ATTR, ATTRv)

ATTRv is transmitted in an autosomal dominant pattern. There are more than 140 pathogenic TTR variants with known propensity for amyloid formation (Table 1). While various variant nomenclatures have been used in the literature, it has been recommended to use the numbering based on the sequence of mature amyloid protein without its 20-amino-acid leader sequence (e.g., Val30Met or V30M, instead of p.Val50Met) [37].

ATTR is a multisystemic disease and the typical clinical phenotype of pathogenic variants of TTR is defined by dominant clinical manifestations and the age of onset. The most common pathogenic variants of TTR represented in the global THAOS registry were Val30Met (V30M), Val122Ile (V122I), Glu89Gln (E89Q), Thr60Ala (T60A) and Ser50Arg (S50R) [27]. The expected age of onset varies between variants and different ethnic groups and geographic areas. Variant V30M, also known as “Portuguese,” may present early with progressive neuropathy and dysautonomia around 20–40 years of age, or late after age 50 with a mixed neuropathic and cardiomyopathic phenotype [38]. The early-onset phenotype of V30M is found mostly in Portugal, while in other endemic populations in Sweden and Japan, patients usually present with a late-onset phenotype with earlier onset of cardiomyopathy and delayed onset of dysautonomia [39]. The age of onset of Portuguese V30M patients was 34, compared with 58.9 years in French V30M patients, while penetrance at age 80 was similar at 91% and 86% [40]. In comparison, penetrance for the same V30M variant in Swedish patients was estimated at 1.7%, 22% and 69% at the ages of 30, 60 and 90 years [41]. The penetrance is reportedly associated with the gender of the parent of origin, with reported higher disease risk and greater anticipation with maternal transmission [42,43]. The most common cardiac variant of ATTRV globally is V122I with high prevalence in western Africans, African Americans and Afro-Caribbeans and late onset of ATTR cardiomyopathy (ATTR-CA) with rare occurrence of significant ATTR neuropathy (ATTR-PN) [44,45]. While V122I probably has the highest global genotype prevalence, it often has a fairly late onset and incomplete penetrance, partly explaining its underrepresentation in the THAOS registry [27]. The penetrance of V122I for ATTR-CA before the age of 75 was estimated at 20%, and for individuals older than 75 at 63% [44]. Variant E89Q usually has a mixed phenotype starting with peripheral neuropathy in the fifth or sixth decade and progressive cardiomyopathy [46]. Variant T60A is often found in individuals of Irish and Scottish descent and typically presents with a mixed phenotype [11]. Variant S50R has been described in an endemic region in Mexico with an early onset of neuropathy, often before age 40, and with a progressive clinical course [47]. Rarely, some of the ATTRv variants may present with oculoleptomeningeal symptoms, including Tyr69His (Y69H), Asp18Gly (D18G), Val30Gly (V30G) and Tyr114Cys (Y114C) [48]. Age at onset of oculoleptomeningeal ATTR (OLMA) is often in the 3rd to 5th decades (20–40 years) [48].

### 4.2. Wild-Type ATTR (ATTRwt)

Wild-type ATTR (ATTRwt), previously known as “senile amyloidosis,” is characterized by late-onset ATTR cardiomyopathy with a normal genotype [49]. The median age of ATTRwt-CA patients is 75 years and 91% are men [49]. The pathogenesis of ATTRwt with normal genotype is not well understood, but circulating TTR in ATTRwt was found to be more thermodynamically unstable and prone to dissociation than in normal controls [50]. Autopsy studies showed cardiac ATTRwt in more than 25% of individuals who died after the age of 85 [51]. It has been estimated (by imaging) that around 6% of cases of HFpEF in older patients are associated with ATTRwt [52]. Cardiomyopathy is the cardinal clinical manifestation of ATTRwt, and neuropathy is typically mild, with only a few reported cases of ATTRwt-neuropathy with histologic confirmation of ATTRwt on nerve biopsies [53]. ATTRwt deposits are found with an increasing frequency with aging, and can be found in connective tissue (tendons, ligaments), lungs, prostate, bone marrow and liver. Wild-type ATTR amyloid deposits are also found mixed with ATTRv deposits in the nerve and in the heart of ATTRv patients [54,55]. ATTRwt disproportionally affects men and affected women are typically older than affected men, but the mechanism of such predilection remains unknown [56].

### 4.3. Acquired ATTR

While liver transplantation has lost its significance as a treatment for ATTR with the introduction of TTR stabilizers and gene silencers, there have been more than 2000 ATTR patients worldwide who received liver transplantation [57]. Explanted livers from ATTR patients have been used for domino liver transplantation for patients with fulminant liver failure. It has been reported that up to 57% of the domino liver transplant recipients may develop acquired ATTRv after a median time of 7.7 years [58]. However, acquired ATTR after domino liver transplantation may develop as early as 2 months after transplantation or as late as 10 years [59].

## 5. Clinical Manifestations of ATTR

Most patients with ATTRv develop multisystemic manifestations of amyloid deposition in various tissues with dominant cardiac, neuropathic or oculoleptomeningeal phenotypes (Table 2) [60]. Clinical symptoms of ATTR are influenced by the distribution of amyloid deposits and are similar to symptoms of other types of amyloidoses, including immunoglobulin light chain amyloidosis.

In ATTRwt, cardiomyopathy is the predominant clinical manifestation in almost all patients. Amyloid deposition and clinical manifestations have also been demonstrated in various other tissues, including musculoskeletal and connective tissue, skin, kidneys, gastrointestinal tissue, lungs and prostate [61]. There is a gender predilection for ATTR phenotypes and cardiac involvement was found more frequently in male ATTR patients in the THAOS registry, while the occurrence of neuropathy was reported as more even between sexes [62].

### 5.1. ATTR Cardiomyopathy (ATTR-CA)

Cardiac phenotype with ATTR-CA is the most common primary clinical manifestation of ATTR and can be associated with various phenotypes. Cardiac ATTR is associated with the accumulation of amyloid deposits in the heart, leading to wall thickening and resulting in impaired systolic function and abnormal global strain [63]. In addition to mechanical impairment of contractility, ATTR also impairs cardiac conduction. ATTR-CA is also associated with an increased risk of atrial fibrillation, atrial flutter and sustained ventricular tachycardia [64]. It has been estimated that up to 69% of patients with ATTR-CA may develop atrial fibrillation [64].

Common ATTRv variants with primary cardiac phenotype include V122I, I68L and L111M (Table 1). Due to the high prevalence of the V122I variant in the Black population, the prevalence of ATTRwt-CA is similar to that of ATTRv-CA associated with the V122I variant [65]. In addition to patients with mixed ATTRv phenotypes, patients with neuropathic variants of ATTR may also subsequently develop ATTR-CA, especially with a later onset of ATTRv [66]. Cardiac phenotype is always the primary clinical manifestation of ATTRwt.

Typical clinical phenotype of ATTR-CA is of restrictive cardiomyopathy with left ventricular hypertrophy and heart failure with preserved ejection fraction (HFpEF) [63]. It has been estimated that up to 12% of HFpEF cases, 10% of heart failure cases with reduced ejection fraction (HFrEF) and 8% of cases with severe aortic stenosis undergoing valve replacement are related to ATTR cardiomyopathy [63]. In clinical practice, ATTR-CA remains significantly underdiagnosed, and in a large cohort of individuals with African ancestry, only 11% of heart failure cases in V122I carriers were previously clinically identified as being caused by V122I ATTR-CA [67]. Additionally, asymptomatic or presymptomatic ATTR-CA identified with abnormal imaging in the absence of heart failure signs is associated with a 23–53% chance of developing heart failure and 1.4–5.1% risk of cardiovascular mortality within 3 years [68].

### 5.2. ATTR Peripheral Neuropathy (ATTR-PN)

ATTR-PN is caused by amyloid deposition in the peripheral nerve and is primarily encountered in ATTRv [69,70,71]. Neuropathy in patients with ATTRwt is typically milder with multiple potential comorbidities and alternative causes [70,72]. Classic clinical features of ATTRv-PN typically include painful axonal sensorimotor polyneuropathy with dysautonomia, but variable phenotypic features have been described in individual patients. In late onset ATTRv-PN, the onset of dysautonomia may be delayed [73]. Late-onset ATTRv-PN is also more likely to have an early involvement of the upper extremities and early loss of vibratory sense and proprioception [39]. When compared to diabetic neuropathy or Charcot–Marie–Tooth disease, ATTRv-PN is typically associated with much faster progression with earlier onset of weakness [34,71,74]. ATTRv-PN can also start as a non-length-dependent neuropathy with early predominant involvement of the upper extremities [75]. Early-onset ATTRv-PN typically starts in the late 20s to early 40s with paresthesia, allodynia or spontaneous pain with reduced sensation to pinprick and cold temperatures distally, and dysautonomia, followed by walking difficulty, loss of vibration sensation and proprioception, and weakness [39]. Clinical features of dysautonomia may include reduced sweating, gastrointestinal dysmotility, orthostatic hypotension and arrhythmias [76]. Gastrointestinal involvement in ATTRv may manifest with dysmotility, abdominal pain, nausea, impaired absorption or weight loss and may have a significant impact on quality of life [77].

Rarely, amyloid deposition may be associated with demyelinating peripheral neuropathy and may be mistaken for chronic inflammatory demyelinating polyneuropathy or Charcot–Marie–Tooth disease [78].

Common ATTRv variants with primary neuropathic phenotype include V30M, S50R and E89Q (Table 1). Similarly to ATTR-CA, in addition to patients with mixed ATTRv phenotype, patients with cardiac variants of ATTR also often subsequently develop ATTR-PN, especially with later onset of ATTRv [66]. It has been estimated that 2.1 to 9.0% of carriers of the most common ATTR-CA variant V122I have polyneuropathy [45]. Neuropathy is a minor clinical manifestation of ATTRwt when present [53,72].

### 5.3. Oculoleptomeningeal ATTR (OLMA)

Most of TTR is synthesized in the liver, but some is also synthesized in the choroid plexus and retina. Accumulation of ATTR in the central nervous system is mostly localized in the leptomeninges and leptomeningeal blood vessels. Clinical manifestations may include “transient focal neurologic events” (TFNE), ischemic stroke or brain hemorrhage, cranial neuropathies, ocular amyloidosis and cognitive decline [48,79]. Other CNS symptoms may include progressive hearing loss, central sleep apnea and hydrocephalus [48,79]. Oculoleptomeningeal ATTR (OLMA) may present early with specified TTR variants, including Tyr69His (Y69H), Tyr114Cys (Y114C), and Asp38Gly (D18G), or may have a delayed onset with dominant neuropathic or cardiac ATTR variants (Table 1) [48,79,80]. The typical onset of OLMA is in the 3rd to 5th decade, followed by rapid progression [48]. ATTR patients who were treated with liver transplantation may develop late manifestations of OLMA at 10–15 years after liver transplantation, as variant TTR production continues after transplantation in the choroid plexus [81]. Currently available TTR gene silencers do not cross the blood–brain barrier, and CNS manifestations of ATTR do not respond to treatment. However, a recent report suggested potential benefits of vutrisiran in some patients with OLMA [82].

Ocular ATTR may lead to various ophthalmologic complications, including vitreous amyloid, glaucoma, dry eye, neurotrophic keratitis, tortuous retinal vessels and scalloped iris [83,84,85]. Abnormal Schirmer test, vitreous amyloidosis and glaucoma were found in 67%, 17% and 20% of V30M patients, respectively [83]. Interestingly, women were more likely than men to have ocular amyloidosis [84].

### 5.4. Connective and Musculoskeletal Tissue ATTR

ATTR deposits have been demonstrated in various types of musculoskeletal tissue, including muscles and tendons, joints and ligaments, manifesting with carpal tunnel syndrome, biceps rupture and spinal stenosis [26]. Carpal tunnel syndrome was recognized early as a systemic manifestation of ATTR [86], and it often precedes dominant ATTR clinical manifestations by 5–10 years [23].) ATTR deposits are often found in ligaments, menisci and articular cartilages, but their clinical significance is mostly uncertain [26,87]. Amyloid deposits in ligamentum flavum can worsen spinal stenosis and may even lead to spinal cord compression [88]. ATTR may also be associated with distal biceps tendon rupture in older patients [89].

### 5.5. Other Clinical Manifestations of ATTR and Multisystemic Amyloid Deposits

While nephropathy is rarely a major clinical feature of ATTR, high prevalence of chronic kidney disease and proteinuria has been described in ATTR patients [90,91]. Decline of kidney function is commonly found in patients with ATTR-CA and is associated with greater mortality and worsened outcomes [90]. ATTR deposits are also commonly found in lung tissue, especially with ATTRwt [92]. Most patients with pulmonary ATTR have concurrent cardiac amyloidosis (82%), but some present with an unexplained respiratory insufficiency [92]. While ATTR in bone marrow is generally considered an incidental finding, in the Mayo cohort of 1469 bone marrow amyloid biopsies, specimens were positive for ATTR in 16.3% of cases, mostly with ATTRwt, which was four times as common as ATTRv [93].

## 6. Natural History

ATTR often follows a rapidly progressive course, and if left untreated, many ATTRv PN patients will deteriorate quickly, with survival of only 5–15 years [74]. Treatment with TTR stabilizers and gene silencers has changed the clinical course by slowing the progression and reducing morbidity and mortality of ATTRv and ATTRwt [94,95,96,97,98,99,100,101].

The progression of ATTRv PN is typically measured by the presence of ambulatory dysfunction and the need for assistive devices. In 1980, Coutinho et al. developed the Familial Amyloid Polyneuropathy (FAP) staging system, which includes three stages [102]. In stage 1, patients can walk unassisted. In stage 2, they need a cane or a walker. In stage 3, they are wheelchair-bound or bedbound [69]. This system can be easily applied in clinical practice and has been used as part of the eligibility criteria for clinical trials of TTR silencers. Studies of untreated disease indicate that each stage lasts approximately 5 years. A 5-year study of patisiran, the first TTR silencer approved for ATTRv PN, demonstrated that treated patients retained their walking ability for longer [96]. During the study, 19.4% of patients still died despite treatment, mostly with advanced polyneuropathy and delayed treatment [96].

Clinical course of ATTR-CM varies depending on the genotype. However, untreated disease is universally fatal. Prior to the introduction of gene silencers and TTR stabilizers in clinical practice, a British study found a median survival from diagnosis in untreated patients at 2.6 years for V122I ATTRv-CM, 4.8 years for ATTRwt-CM, and 5.8 years for non-V122I ATTRv-CM [103]. Age, genotype, disease stage, and left ventricular ejection fraction were independently associated with survival, emphasizing the importance of early diagnosis [103].

A recent meta-analysis noted a higher 2-year survival rate for ATTRwt compared to ATTRv. For the latter, patients with V122I and T60A variants had shorter survival compared to patients with V30M variants [104]. Among patients with cardiac amyloidosis, worsening heart failure is the most common cause of death, accounting for 79% of cardiovascular deaths in ATTRv and 76% in ATTRwt. Infections are the most common non-cardiovascular cause of death in this patient population [105].

## 7. Asymptomatic and Presymptomatic ATTR and Management of ATTRv Variant Carriers

Technological advances and increased access to amyloid imaging and genetic testing have facilitated the identification of an unprecedented number of asymptomatic or presymptomatic ATTR patients and ATTRv gene variant carriers. Genetic counseling plays an important role in diagnostic testing, information on reproductive options and presymptomatic testing for presymptomatic relatives [69]. Country-specific guidelines for ATTR-related genetic testing should be considered, depending on geographic location and predominance of local TTR variants [69]. Genetic counseling of asymptomatic individuals is usually done through genetics, and diagnostic testing is usually guided by an ATTR clinical specialist [69]. ATTRv is an autosomal dominant disease, and most affected patients have monoallelic pathogenic variants. Most of the ATTRv variants have incomplete penetrance, and the onset and clinical manifestations vary even within the same family. Homozygous biallelic TTR variants have variable impact, with some individuals showing earlier onset and rapidly progressive course, and others with late onset and slow progression with the same variant [106,107]. The available treatments for ATTRv typically slow down or arrest the progression of peripheral neuropathy and cardiomyopathy and are more effective if started early. Reversal of ongoing symptoms is less likely. However, the optimal timing of the start of the ATTR treatment remains unclear and some of the clinical manifestations of ATTR, like carpal tunnel syndrome, often precede other clinically impactful manifestations (e.g., cardiomyopathy, peripheral neuropathy) by 5–10 years or even longer. Careful evaluations and monitoring of asymptomatic gene carriers are needed to promptly diagnose early manifestations of ATTRv affecting the heart and peripheral nervous system and initiate the appropriate treatment [108]. Evaluation of ATTRv V30M carriers with normal nerve conduction studies showed reduced skin innervation (consistent with small fiber neuropathy) in 89% of subjects and cardiac involvement in 13–16% by bone scintigraphy [108].

In patients with asymptomatic ATTR-CA, there is a clinical decision after the incidental discovery of cardiac ATTR, whether patients need to be treated immediately or should be closely monitored. As some of the patients may quickly become symptomatic, these patients need to be followed closely and early treatment with disease-modifying medications should be considered [109].

## 8. Diagnosis of ATTR

Early and accurate diagnosis of ATTR improves treatment benefits and reduces morbidity. Tissue amyloidosis can be diagnosed histopathologically and noninvasively by imaging with bone-avid radiotracers and amyloid PET tracers [110]. Cardiac MRI may also reveal characteristic findings suggestive of cardiac amyloidosis [111]. Genetic testing may confirm the carrier status of relatives of ATTR patients or may reveal newly found TTR variants during the workup of various clinical manifestations.

Histopathologic diagnosis of amyloidosis is typically based on positive Congo red tissue staining [112]. Tissue diagnosis can be obtained on a biopsy specimen of the clinically involved tissue (e.g., heart, peripheral nerve), fat pad biopsy, other diagnostic biopsies (e.g., bone marrow, gastrointestinal or salivary gland) or skin biopsy [112]. However, Congo red staining does not distinguish ATTR from other types of amyloidosis and amyloid typing is needed to determine the underlying disease. Clinical phenotypes of ATTR-CA and ATTR-PN cannot be distinguished from light chain amyloidosis and accurate amyloid typing is essential for correct diagnosis and effective management. Proteomics, combining liquid chromatography and mass spectrometry, is preferred to immunohistochemistry (when available) to confirm the type of amyloidosis [112,113].

Noninvasive diagnosis of cardiac TTR amyloidosis has been greatly facilitated with the use of cardiac scintigraphy with bone-avid radiotracers, as abnormal testing is relatively specific for ATTR, especially in the absence of gammopathy [114]. Additionally, cardiac scintigraphy is widely available and most commonly used bone-avid radiotracers include ^99m^Tc-hydroxymethylene diphosphonate (^99m^Tc-HMDP) and ^99m^Tc-pyrophosphate (^99m^Tc-PYP) [115].

The use of amyloid PET tracers allows diagnosing amyloid deposits in various tissues and organs but current tracers (including ^18^F-florbetapir, ^18^F-florbetaben, ^18^F-flutemetamol and ^11^C-Pittsburgh compound B) are not specific for ATTR [81,116,117]. Whole-body PET with ^18^F-florbetapir may detect amyloid infiltration in various tissues and organs before clinical signs of organ involvement [118]. Most recently, another peptide-based pan-amyloid tracer, ^124^I-evuzamitide, has been introduced as a more sensitive tracer allowing early diagnosis of amyloid deposition [119,120]. Radiolabeling of peptide p5+14 with either iodine-124 or technetium-99m has been used for PET/CT (^124^I-evuzamitide) or SPECT/CT radiotracers to detect different types of amyloid and even identify them as the imaging characteristics are uniquely different for each type [121].

Cardiac amyloidosis is associated with late gadolinium subendocardial or transmural enhancement, but the same findings can be seen with non-TTR amyloidoses, and endomyocardial biopsy may be needed to determine the type of amyloid [122]. MR neurography may detect and quantify nerve injury in ATTR-PN, but the imaging results are not specific for peripheral nerve amyloidosis [123].

While late-onset ATTR-CA is most frequently caused by ATTRwt, one of five patients with late-onset ATTR-CA still has ATTRv [21]. Genetic testing is recommended for all adult ATTR patients to distinguish ATTRv from ATTRwt [21].

## 9. Treatment of ATTR

Tissue injury in amyloidoses is caused by amyloid deposits in various tissues which interfere with their physiologic functions and may be toxic. Potential treatment strategies are based on reducing the production of amyloid by stabilization of transthyretin and by reducing the circulating levels of transthyretin protein by gene silencing and by facilitation of the removal of the existing amyloid deposits (Table 3) [124,125].

The first treatment with proven benefits for patients with ATTRv was liver transplantation with replacement of the native liver producing variant TTR protein with a liver allograft producing wild-type TTR protein [57]. Complex post-transplant clinical course and development of other treatment options limited the role of liver transplantation in the treatment of ATTR. Subsequent clinical trials demonstrated clinical benefits of TTR stabilizers and gene silencers for the treatment of both ATTRv-PN and ATTR-CA, although their approval status and availability for treatment of ATTRv-PN and ATTR-CA vary in different countries and regions. In the US, only gene silencers are approved for the treatment of ATTRv-PN. In Europe, Latin America and Japan, tafamidis is also approved for the treatment of early stages of ATTRv-PN. For treatment of ATTR-CA, vutrisiran is approved in the US and EU, in addition to TTR stabilizers (tafamidis, acoramidis). In Japan and Brazil, only TTR stabilizers are used for ATTR-CA, and in China, tafamidis is currently the only medication approved for ATTR.

Current treatment of ATTR-CA and ATTRv-PN is based on transthyretin protein stabilization and gene silencing, and liver transplantation has taken the role of second-line treatment for ATTR [69,71]. Experimental treatments facilitating amyloid removal and TTR gene editing are explored as well.

Another ongoing challenge is the treatment of other ATTR complications, including OLMA, pulmonary and kidney ATTR, but the patients with pulmonary and kidney amyloidosis usually manifest ATTR-CA as well. Currently available treatments are not considered effective for OLMA, although tafamidis does cross the blood–brain barrier, and there was a recent report of improvement of OLMA after treatment with vutrisiran (subcutaneously) [82,126].

### 9.1. Liver Transplantation

Orthotopic liver transplantation (OLtx) was the first treatment shown to slow down the progression of ATTRv [57]. Transplant recipients may develop post-transplant ATTRwt cardiomyopathy (and neuropathy) and leptomeningeal ATTR [81]. Better outcomes are reported in patients with early-onset V30M mutation than with late-onset V30M or non-V30M ATTR [127]. Explanted liver from ATTRv patients may be used for domino liver transplantation for patients with liver failure, but the allograft recipients may later develop iatrogenic or acquired ATTR [59]. Liver re-transplantation at early stages of acquired ATTR may improve outcomes [128]. In addition to liver transplantation, heart transplantation has been used for the treatment of heart failure caused by ATTR cardiomyopathy, by itself or in combination with liver or kidney transplantation [129]. While the role of liver transplantation for treatment of ATTRv is now diminished with the availability of modifying therapies, heart transplantation remains an option for treatment of ATTR patients with advanced heart failure [130].

### 9.2. Transthyretin Stabilization

Dissociation of TTR tetramers is the rate-limiting step in the formation of ATTR amyloid fibrils [131]. TTR tetramer dissociation leads to the formation of TTR dimers and monomers, and once TTR monomers misfold, they can no longer return to the tetramer state and proceed to aggregate, leading to amyloid fibril formation. Kelly and his group have found that the binding of T4 at its site on TTR stabilizes the tetramer and inhibits TTR amyloid formation [132]. This has been utilized by all currently available TTR stabilizers as they all bind to the T4 site on the TTR molecule [131]. In clinical practice, TTR stabilizers, including tafamidis, diflunisal and acoramidis, have all been shown to slow the progression of ATTR. Tafamidis and diflunisal showed benefits with both cardiomyopathy and peripheral neuropathy [98,101,133,134], and acoramidis showed benefits in the treatment of cardiomyopathy [100]. Tafamidis reduced all-cause mortality and cardiovascular hospitalization over 30 months in ATTR-CA by 30% and 32%, respectively [101]. The benefits of tafamidis for ATTR-PN have been relatively mild, with slowing of progression of neuropathy in its early stages, and it has been used in Europe, Japan and Brazil for treatment of mild or early ATTRv-PN [135,136]. Patients with ATTRv-PN treated with low-dose tafamidis (20 mg daily) had significantly less weakness, but the trend of improvement of quality of life and NIS-LL score was not statistically significant at 18 months [98]. While tafamidis also crosses the blood–brain barrier and is found in potentially effective concentrations in cerebrospinal fluid, the attempts to use it for the treatment of leptomeningeal ATTR have not been effective [137].

Diflunisal is a cheap alternative to other TTR stabilizers and it did demonstrate mild benefits for patients with ATTRv-PN and ATTR-CA [133]. The use of diflunisal is limited by the potential side effects of long-term use of non-steroidal anti-inflammatory medications in patients with cardiomyopathy and the potential nephrotoxicity.

Most recently, another TTR stabilizer, acoramidis, showed a reduction in cardiovascular hospitalizations and all-cause mortality in patients with ATTRwt and ATTRv cardiomyopathy by 31% and 59%, respectively [138].

The ongoing study of acoramidis in asymptomatic ATTRv variant carriers may expand the use of stabilizers for ATTR prevention [139].

### 9.3. Gene Silencing of TTR

Formation of amyloid in ATTR is also limited by the availability of circulating TTR protein. Gene-silencing therapies are based on the suppression of the expression of pathogenic gene variants, together with wild-type variants, without altering DNA. Gene-silencing treatments based on small interfering RNA (siRNA) and antisense oligonucleotides (ASO) promote degradation of mRNA or change splicing patterns [140]. Gene silencers reduce the production of TTR in the liver by selectively targeting the endogenous receptor expressed by hepatocytes to facilitate intracellular delivery of these nucleic-acid-based drugs [141]. Currently approved gene silencing therapies for ATTR include patisiran and vutrisiran (siRNA) and eplontersen (ASO). After inotersen (ASO) was approved for ATTRv-PN following patisiran in 2018, its clinical use was limited by the stringent requirements for monitoring for possible thrombocytopenia and glomerulonephritis, and it has been substituted with chemically modified and safer medication, eplontersen [97,99]. All gene silencers have shown remarkable efficacy in the treatment of ATTRv-PN and provide sustained 75–85% reduction in serum TTR levels [94,95,97,99]. Treatment of ATTRv-PN with different gene silencers had almost uniformly stabilized measures of neuropathy progression (mNIS+7) and showed better quality of life when compared to placebo groups [94,95,97]. Vutrisiran is also approved for the treatment of ATTR-CA, as it reduced the risk of cardiovascular events by 28–33% and mortality of all causes at 42 months by 35% [142].

Currently available gene silencing therapies do not cross the blood-brain barrier and are generally not considered as effective treatments for OLMA, although a recent report by Rossi suggests potential benefits of gene silencing for some patients with CNS ATTR [82].

Combination of gene-silencing therapy with TTR stabilizers has not been systematically studied as a treatment for ATTR, although some patients in clinical practice are treated with such dual therapy. Clinical benefits of this costly combination of these two classes of medications may be impeded by the limited amounts of TTR (15–25% of pretreatment levels) available for interaction with stabilizing agents. A small number of patients in clinical studies of ATTRv-PN who were treated both with TTR stabilizers and gene silencers showed similar outcomes as the patients treated with gene silencers only [143]. Gene-silencing treatments for non-ambulatory patients with advanced amyloidosis may not offer the same benefits as with milder disease but their use is still recommended [71].

### 9.4. Gene Editing of TTR

Another approach for targeted gene silencing used CRISPR genome editing to achieve sustained gene knockdown [144]. Gene editing with CRISPR-Cas9, aiming at selective inactivation of TTR production in the liver, has been used as an investigational therapy for ATTR [145,146]. In a phase I study involving patients with ATTR-CA, nexiguran ziclumeran (nex-z; previously known as NTLA2001) was well tolerated and led to a 90% reduction in TTR levels at 12 months after a single dose [145]. Selective suppression of TTR production in patients with ATTRv-PN by 92% was maintained for 24 months, resulting in stabilization of ATTRv-PN in most of the patients treated [145], and the ongoing study should demonstrate the extent of benefits and the safety of this approach.

### 9.5. Anti-Amyloid Therapies

The use of TTR stabilizers and gene silencing does not have a direct impact on already formed amyloid tissue deposits, but recent reports of endogenous antibody-mediated amyloid removal prompted additional attention to amyloid removal treatment options [147]. Currently, there are several ongoing clinical trials investigating experimental treatments of ATTR with humanized and recombinant human anti-amyloid antibodies [148,149]. A recent report also suggested that treatment with TTR stabilizers may enhance endogenous amyloid-removing antibodies [150]. Additionally, antibody-peptide fusion protein AT-02 is investigated as a possible treatment that would promote the removal of various types of amyloid [119].

## 10. Future Perspectives

The future of management of ATTR is moving towards an individualized approach required by a wide range of clinical manifestations and phenotypes. Clinical studies have demonstrated the benefits of early treatment before advanced clinical manifestations become irreversible. Recent advances in genetic testing have facilitated identification of asymptomatic carriers and treatment with disease-modifying medications is initiated with the onset of clinical manifestations based on a combination of clinical symptoms, abnormal test results and evidence of amyloid deposition [108].

Clinical studies that led to the approval of TTR stabilizers and gene silencers for treatment of ATTR demonstrated reduction in hospitalization rates and mNIS+7 score declines in treated patients [94,95,97,100,101], but these measures are difficult to use in daily clinical practice. Plasma neurofilament light chain (Nfl) in ATTRv-PN, and NT-proBNP and troponin T in ATTR-CA, have established themselves as biomarkers associated with disease progression and severity [110,151].

The effectiveness of TTR suppression is proven by serum TTR (prealbumin levels). Gene-silencing and gene-editing treatments have demonstrated sustained reduction in transthyretin (prealbumin) levels by 75–85%, but it remains unclear what would be the optimal reduction level and whether it should be individualized depending on TTR variants or other clinical features. Physiologic consequences of long-term depletion of transthyretin and their impact on thyroid function and visual function are uncertain. The role of following TTR levels in clinical practice has not been defined, but these levels may be helpful to confirm TTR reduction in patients treated with gene silencers. Treatment with TTR stabilizers may lead to an increase in TTR levels which may predict reduced risk of cardiovascular events [152].

Immediate risks of gene editing include hepatotoxicity and immune response. Long-term effects of gene editing are still not well understood but future potential challenges include the risk of genome-wide off-target effects, genotoxicity and immune response [153]. Gene editing may lead to unintended changes in gene expression, translocations and chromosome rearrangements, and may require continuous surveillance and strict quality control. Future improvements may also allow tissue-specific gene editing targeting key organs (e.g., liver, retina, choroid plexus). Early clinical trials of ocular disorders suggest that gene editing may become a potential option for the treatment of OLMA as well [154].

Economic burden of ATTR includes diagnostic evaluations (e.g., imaging, genetic testing, biopsies), hospitalizations and outpatient evaluations, loss of economic productivity for patients and their caregivers and cost of supportive and pharmacologic therapies. Clinical benefits of ATTR therapies and reduced hospitalizations may reduce the economic burden of the disease, but current therapy prices do not meet cost-effectiveness thresholds [155]. The cost of treatment of ATTR in individual patients remains very high and the economic impact of ATTR treatment in endemic regions may significantly impact local healthcare spending. Additionally, a cumulative cost of treatment of the common variant of ATTRwt may drain resources in non-endemic regions as well.

## 11. Conclusions

Transthyretin amyloidosis is a progressive multisystemic disease with a wide spectrum of clinical manifestations, high morbidity and mortality if left untreated. ATTR presents as an autosomal dominant disease in its hereditary form due to pathogenic mutations or as a “wild-type ATTR” (previously known as “senile amyloidosis”) with a normal genotype and late onset. Cardinal clinical manifestations include cardiomyopathy and peripheral neuropathy, but many other clinical manifestations include oculoleptomeningeal amyloidosis, dysautonomia, nephropathy, carpal tunnel syndrome, distal biceps tendon rupture and spinal stenosis. Current treatment is based on TTR stabilizers and RNA-based gene silencers, and ongoing studies are also exploring the use of anti-amyloid treatments and gene editing. A wide spectrum of clinical manifestations warrants a multidisciplinary approach to management and prevention of complications. Early diagnosis and treatment initiation before irreversible organ damage improve treatment outcomes. Current treatments are still not effective for the treatment of ocular and CNS ATTR, and the development of new treatments is needed. Future challenges will also include providing access to effective ATTR treatments to all affected patients in various geographic regions.

## Figures and Tables

**Table 1:** ATTR variants and their typical clinical phenotypes.

	Clinical Manifestations	Age of Onset	Geographic Area
L12P	CA, PN, OLMA	20–40	Great Britain
D18G	OLMA, CA	20–50	Hungary
A25T	OLMA, PN	40–50	Japan
V30M	CA, PN, OLMA	early—20–40 (Por); late—>50 (Jpn, Swe)	Portugal, Sweden, Japan
S50R	PN, CA	30–45	Mexico
T60A	CA, PN	40–80	Ireland, United Kingdom, USA
F64L	PN, CA	50–70	Italy, USA
I68L	CA, PN	50–70	Italy, Germany, USA
S77Y	CA, PN	50–60	France, Germany, USA
E89Q	PN, OLMA	50–60	Bulgaria, Italy
L111M	CA	40–70	Denmark
Y114C	OLMA, PN, CA	30–40	Japan, China
V122del	CA, OLMA	30–60	Ecuador, Spain
V122I	CA, PN, OLMA	60–70	Western Africa, the Caribbean, USA

CA—cardiomyopathy, PN—peripheral neuropathy, OLMA—oculoleptomeningeal.

**Table 2:** Multisystemic manifestations of ATTR.

Clinical Syndrome	Symptoms
Cardiomyopathy	Fatigue, peripheral edema, dyspnea
Peripheral neuropathy	Sensory loss, weakness, paresthesias, dysautonomia
Ocular amyloidosis	Blurry vision, loss of vision
Leptomeningeal amyloidosis	Microhemorrhage, “transient neurologic events”, seizures, cognitive decline
Connective tissue amyloidosis	Entrapment neuropathies, spinal stenosis, biceps tendon rupture
Nephropathy	Proteinuria, peripheral edema, kidney insufficiency
Gastrointestinal amyloidosis	Dysmotility, nausea, abdominal pain, malabsorption, weight loss
Pulmonary amyloidosis	Dyspnea

**Table 3:** Clinical and investigational treatments for ATTR.

Class	Name	Mechanism	Treatment of CA	Treatment of PN
TTR stabilizer	Tafamidis	TTR stabilizer	US, EU, JPN, CHN, BRA	EU, JPN, BRA
	Acoramidis	TTR stabilizer	US, EU, JPN	-
	Diflunisal	TTR stabilizer	Off label	Off label
Gene silencer	Patisiran	siRNA	-	US, EU, JPN
	Inotersen	ASO	-	US, EU
	Vutrisiran	siRNA	US, EU	US, EU, JPN
	Eplontersen	ASO	-	US, EU, CHN
Gene editing *	Nexiguran ziclumeran	CRISPR-Cas9	Experimental	Experimental

*—experimental treatment.

## Data Availability

No new data were created or analyzed in this study.

## Abbreviations

The following abbreviations are used in this manuscript:

ATTR	Transthyretin amyloidosis
ATTR-CA	Transthyretin amyloid cardiomyopathy
ATTRv-PN	Transthyretin peripheral neuropathy
ATTRv	Variant transthyretin amyloidosis
ATTRwt	Wild-type transthyretin amyloidosis
OLMA	Oculoleptomeningeal amyloidosis
PMPY	Per million person-years
